# The alternative respiratory pathway is involved in brassinosteroid-induced environmental stress tolerance in *Nicotiana benthamiana*

**DOI:** 10.1093/jxb/erv328

**Published:** 2015-07-14

**Authors:** Xing-Guang Deng, Tong Zhu, Da-Wei Zhang, Hong-Hui Lin

**Affiliations:** ^1^Ministry of Education Key Laboratory for Bio-Resource and Eco-Environment, College of Life Science, State Key Laboratory of Hydraulics and Mountain River Engineering, Sichuan University, Chengdu, 610064, PR China; ^2^Chengdu Institute of Biology, Chinese Academy of Sciences, Chengdu, 610041, PR China

**Keywords:** Alternative oxidase, brassinosteroids, *Nicotiana benthamiana*, reactive oxygen species, stress tolerance.

## Abstract

Brassinosteroids (BRs), plant steroid hormones, play essential roles in modulating cell elongation, vascular differentiation, senescence, and stress responses. However, the mechanisms by which BRs regulate plant mitochondria and resistance to abiotic stress remain largely unclear. Mitochondrial alternative oxidase (AOX) is involved in the plant response to a variety of environmental stresses. In this report, the role of AOX in BR-induced tolerance against cold, polyethylene glycol (PEG), and high-light stresses was investigated. Exogenous applied brassinolide (BL, the most active BR) induced, while brassinazole (BRZ, a BR biosynthesis inhibitor) reduced alternative respiration and *AOX1* expression in *Nicotiana benthamiana*. Chemical scavenging of H_2_O_2_ and virus-induced gene silencing (VIGS) of *NbRBOHB* compromised the BR-induced alternative respiratory pathway, and this result was further confirmed by *NbAOX1* promoter analysis. Furthermore, inhibition of AOX activity by chemical treatment or a VIGS-based approach decreased plant resistance to environmental stresses and compromised BR-induced stress tolerance. Taken together, our results indicate that BR-induced AOX capability might contribute to the avoidance of superfluous reactive oxygen species accumulation and the protection of photosystems under stress conditions in *N. benthamiana*.

## Highlight

RBOHB-dependent ROS production is required for BR-induced AOX capability and the activated AOX contributes to the enhancement of environmental stress tolerance in *N. benthamiana*.

## Introduction

During growth and development, plants have to cope with a variety of biotic (i.e. viruses, bacteria, fungi, and insects) and abiotic (i.e. drought, salt, wounding, and changes in temperature and light) stresses. To survive such environmental stress, plants have developed elaborate mechanisms to perceive external signals and manifest adaptive responses to environmental changes. Phytohormones and reactive oxygen species (ROS) play crucial roles in the regulation of multiple plant responses to a variety of stresses (Baxter *et al.*, 2013; Alazem and Lin, 2014). Moreover, there is also evidence to suggest that the mitochondrial alternative respiratory pathway is involved in the adaption of plants to various environmental stresses (Hanqing *et al.*, 2010; Vanlerberghe, 2013; Dahal *et al.*, 2014; Kühn *et al.*, 2015).

Brassinosteroids (BRs) are a class of steroid phytohormone that regulate many aspects of plant growth and development (De Bruyne *et al.*, 2014; Wang *et al.*, 2014). The BR signalling pathway is well established (Yang *et al.*, 2011; Choudhary *et al.*, 2012; Guo *et al.*, 2013). In plants, BRs are perceived by the plasma-membrane-localized and leucine-rich-repeat receptor kinase BR insensitive 1 (BRI1) (Li and Chory, 1997). Another leucine-rich-repeat receptor, the somatic embryogenesis receptor kinase 3 (SERK3), also named BRI1-associated kinase 1 (BAK1), physically interacts with BRI1 and plays an essential role as an enhancer in BR signalling (Li *et al.*, 2002; Nam and Li, 2002). BRs signal through receptor kinase BRI1, co-receptor BAK1, and several other signalling components to control BRI1 EMS suppressor 1 (BES1) and brassinazole resistant 1 (BZR1) family transcription factors that modulate thousands of target genes (Wang *et al.*, 2002; Yin *et al.*, 2002; Yu *et al.*, 2011; Zhang *et al.*, 2014*a*). In addition to their pivotal role in plant growth and development, BRs appear to protect plants from a variety of environmental stresses. There have been several reports describing the relationship between BRs and abiotic stress responses such as high or low temperature, drought, salinity, and heavy metal contamination (Krishna, 2003; Kagale *et al.*, 2007; Bajguz and Hayat 2009; Xia *et al.*, 2009; Jiang *et al.*, 2013). Several recent studies have also revealed that BRs are involved in the pathogen defence response (Heese *et al.*, 2007; Belkhadir *et al.*, 2012; Nahar *et al.*, 2013). However, the mechanisms by which BRs enhance plant stress tolerance have so far remained largely unknown.

The plant response to various types of stresses is associated with the generation of ROS (Baxter *et al.*, 2013). Many studies show that ROS, especially H_2_O_2_ generated by NADPH oxidases encoded by respiratory burst oxidase homolog (*RBOH*) genes, play important roles in the plant response to biotic and abiotic stresses (Yoshioka, 2003; Torres and Dangl, 2005; Marino *et al.*, 2012; O’Brien *et al.*, 2012). In *Arabidopsis*, *rbohD* and *rbohE* mutants show decreased ROS production in response to infection with virulent *Pseudomonas syringae* pv. *tomato* DC3000 (Kwak *et al.*, 2003). Silencing *RBOHA* and *RBOHB* in *N. benthamiana* plants reduced ROS production and compromised resistance to *Phytophthora infestans* (Yoshioka, 2003). Meanwhile, ROS also function as a second messenger in phytohormone signalling and other important biological processes (Desikan *et al.*, 2004; Xia *et al.*, 2009; Zhou *et al.*, 2014*a*). To utilize ROS as signalling molecules, non-toxic levels must be maintained in a delicate balancing act between ROS production and scavenging pathways.

Mitochondrial respiration provides the energy necessary to drive cellular metabolism and transport processes. Besides the cyanide (CN)-sensitive cytochrome pathway of mitochondrial electron transport, plants contain a CN-resistant alternative pathway (Vanlerberghe, 2013). It is well known that the alternative respiratory pathway is catalysed by the alternative oxidase (AOX), which is located in the mitochondrial inner membrane and acts as a terminal oxidase in the plant mitochondrial electron transport chain (Siedow and Umbach 1995; Arnholdt-Schmitt *et al.*, 2006). AOX branches from the main respiratory chain and leads to the release of energy as heat (Millenaar and Lambers 2003). Studies on functions of the alternative pathway in plants have been carried out for many years, and AOX has been established as one of the essential defence components in the plant response to environmental stress (Van Aken *et al.*, 2009; Kühn *et al.*, 2015). These include abiotic stresses such as drought (Bartoli *et al.*, 2005; Dahal *et al.*, 2014), high salt (Wang *et al.*, 2010), and low temperature (Ederli *et al.*, 2006; Wang *et al.*, 2012), as well as biotic stresses such as infection by pathogens (Fu *et al.*, 2010; Cvetkovska and Vanlerberghe, 2012; Liao *et al.*, 2012). Several lines of evidence, particularly those results obtained with transgenic tobacco and *Arabidopsis*, have suggested that AOX may serve a general function by limiting the formation of mitochondrial ROS (Wang *et al.*, 2012; Panda *et al.*, 2013). In addition, recent studies have shown that AOX is also involved in optimizing photosynthesis, and it may have a particular role in relieving the over-reduction of chloroplasts (Yoshida *et al.*, 2008; Zhang *et al.*, 2010, 2014*b*).

To date, few reports in the literature have focused on the effects of BRs on mitochondria and the relationship between AOX and BR signalling pathway in the process of plant metabolism, especially under stress conditions. Some previous studies showed that elevation of BR levels resulted in increased production of H_2_O_2_ via RBOHs (Xia *et al.*, 2009; Zhang *et al.*, 2010), which in turn play a crucial role in stress tolerance against a subset of abiotic stresses. In addition, it has been demonstrated that ROS might be involved in the stress-induced increase of the alternative respiratory pathway (Wagner, 1995; Neill *et al.*, 2002). Therefore, we hypothesized that there might be a link between AOX and BR signalling during the induction of plant stress tolerance. Here, this hypothesis was tested and our experimental results demonstrated that AOX was involved in BR-induced stress tolerance. Furthermore, the possible relationship between AOX and BR signalling in alleviating stress-induced damage was investigated.

## Materials and methods

### Plant materials and growth conditions

*N. benthamiana* plants were grown in a greenhouse at 25 °C and with cycles of 16h of light (100 μmol m^–2^ s^–1^) and 8h of darkness. Seedlings used in the experiments were 5–6 weeks old. For environmental stress tolerance measurement, plants were exposed to cold stress (4 °C), polyethylene glycol (PEG) stress [16% PEG 6000 (w/v) solution] and high-light (HL) stress (600 μmol m^–2^ s^–1^) for 3 d.

### Chemical treatments

Brassinolide (BL, the most active BR) and brassinazole (BRZ, a specific inhibitor of BR biosynthesis) were purchased from Wako Pure Chemical Industries (Chuo-Ku, Osaka, Japan) and Santa Cruz Biotechnology (Dallas, Texas, USA), respectively. Salicylhydroxamic acid (SHAM, an inhibitor of the AOX pathway) and dimethylthiourea (DMTU, an H_2_O_2_ scavenger) were purchased from Sigma (St Louis, USA). The hormone and inhibitor solutions were prepared in distilled water containing 0.02% (v/v) Tween 20. The chemicals and the concentrations used are as follows: BL, 0.01, 0.1, 1, and 5 μM; BRZ, 1 μM; SHAM, 1mM; DMTU, 5mM. Distilled water containing 0.02% (v/v) Tween 20 was used as a control treatment. For SHAM+BL treatment, plants were first sprayed with 1mM SHAM, and 8h later were sprayed with 0.1 μM BL for another 24h. For DMTU+BL treatment, plants were first sprayed with 5mM DMTU, and 8h later were sprayed with 0.1 μM BL for another 24h. The plants were then exposed to environmental stress.

### Tobacco rattle virus (TRV)-mediated virus-induced gene silencing (VIGS) assay

VIGS was performed as described (Zhu *et al.*, 2014). For construction of VIGS vectors, partial cDNA of *NbAOX1* (281bp), *NbRBOHA* (278bp), and *NbRBOHB* (365bp) was amplified by reverse transcription (RT)-PCR from a cDNA library of *N. benthamiana* leaf tissues using gene-specific primers (Supplementary Table S1, available at *JXB* online). These PCR products were then cloned into the TRV vector (pTRV2). For the VIGS assay, pTRV1 or pTRV2 (with the inserted fragment) were introduced into *Agrobacterium tumefaciens* GV2260. A mixture of equal parts of *Agrobacterium* cultures containing pTRV1 and pTRV2 or its derivatives was inoculated into four-leaf-stage plants. To determine the efficiency of VIGS, quantitative real-time PCR was performed with primers targeting sites outside the cloned fragments in the upper leaves at 12 d post-inoculation.

### Respiration measurements

Respiratory oxygen consumption was measured using Clark-type electrodes (Hansatech, King’s Lynn, UK) as described previously (Xu *et al.*, 2012). In brief, approximately 50mg of leaves was cut into small pieces and pre-treated with 5ml of deionized water for 15min in order to eliminate wound-induced respiration. Measurements were done at 25 °C in a final volume of 2ml of phosphate buffer (pH 6.8), and the cuvette was tightly closed to prevent diffusion of oxygen from the air. The total respiration (*V*_t_) was defined as O_2_ uptake rate by *N. benthamiana* leaves. Next, 1mM KCN was added to obtain the O_2_ uptake rate defined as *V*_0_. The residual respiration (*V*_r_) was estimated by measuring the rate of O_2_ uptake in the presence of both 1mM KCN and 0.5mM *n*-propyl gallate. The capacity of the cytochrome pathway (*V*_cyt_) and the alternative pathway (*V*_alt_) was calculated as *V*_cyt_=*V*_t_ – *V*_0_ and *V*_alt_ *=V*_0_ – V_r_.

### RNA extraction, RT-PCR and quantitative real-time PCR

Total RNA was extracted using Trizol reagent (Invitrogen, Shanghai, China) from *N. benthamiana* leaves according to the manufacturer’s recommendations. All RNA samples were treated with DNase I before PCR. For RT-PCR, the first-strand cDNA was prepared using Moloney murine leukemia virus reverse transcriptase (Invitrogen). To further assay the expression levels of genes, quantitative real-time PCR analysis was performed on an iCycler (Bio-Rad, Beijing, China). Relative quantitation of the target gene expression level was performed using the comparative *C*_t_ (threshold cycle) method (Xu *et al.*, 2012). At least three biological replicates were performed for each sample, and three technical replicates were analysed for each biological replicate. Amplification of the *Actin* gene was used as an internal control. The primer sequences are shown in Supplementary Table S1.

### Characterization of promoter activity

*N. benthamiana AOX1* promoter sequences were defined as 1150bp sequences upstream of the translation start codon and were downloaded from the *N. benthamiana* genome database (http://solgenomics.net/organism/nicotiana-benthamiana/genome). ROS-response elements in the promoter were analysed with the sequences indicated in Ho *et al.* (2008) and Petrov *et al.* (2012).

To determine the promoter activity, the whole region and truncated fragments of the promoter region were amplified using specific primers (Supplementary Table S2, available at *JXB* online) and fused independently to the the β-d-glucuronidase (GUS) or luciferase (LUC) reporter gene in the pBI121 vector. Additionally, the cauliflower mosaic virus (CaMV) 35S promoter was fused to GUS or LUC as a control for variation in transformation rate. All constructs were transformed into *Agrobacterium* strain EHA105. Transient expression was analysed in *N. benthamiana* leaves as described previously (Deng *et al.*, 2015). After 12h of transient transformation, the leaves were treated with chemicals. Three days after infiltration, GUS and LUC activity was determined as described previously (Ho *et al.*, 2008). For GUS staining, inoculated leaves were infiltrated with a solution containing 5mg ml^−1^ of 5-bromo-4-chloro-3-indolyl β-d-glucuronide as the substrate in a buffer containing 100mM phosphate buffer (pH 7.0), 0.5mM potassium ferrocyanide, 0.5mM potassium ferricyanide, 10mM EDTA and 0.3% (v/v) Triton X-100, and incubated at 37 °C.

### Analysis of chlorophyll fluorescence

Chlorophyll fluorescence was determined with an imaging pulse amplitude modulated fluorometer (IMAG-MINI, Heinz Walz, Germany). For measurement of *F*_v_/*F*_m_, plants were dark adapted for 30min. Minimal fluorescence (*F*_o_) was measured during the weak measuring pulses, and maximal fluorescence (*F*_m_) was measured by a 0.8 s pulse of light at about 4000 μmol m^–2^ s^–1^. An actinic light source was then applied to obtain steady-state fluorescence yield (*F*_s_), after which a second saturation pulse was applied for 0.7 s to obtain light-adapted maximum fluorescence (*F*_m_ *’*). *F*_v_ */F*_m_ and non-photochemical quenching (NPQ) were calculated as *F*_m_ – *F*_o_/*F*_m_ and (*F*_m_/*F*_m_ *’*) – 1, respectively.

### Superoxide and H_2_O_2_ staining

Superoxide and H_2_O_2_ staining were visually detected with nitro blue tetrazolium (NBT) and 3,3′-diaminobenzidine (DAB). *N. benthamiana* leaves were vacuum infiltrated with NBT (0.5mg ml) solutions for 2h or DAB (2mg ml^–1^) solutions for 8h. Leaves were then decolorized in boiling ethanol (95%) for 15min.

### NADPH oxidase activity and H_2_O_2_ determinations

For the determination of NADPH oxidase activity, leaf plasma membranes were isolated using a two-phase aqueous polymer partition system (Larsson *et al.*, 1987). The NADPH-dependent superoxide generating activity was determined as described previously (Xia *et al.*, 2009). H_2_O_2_ accumulation was determined using an Amplex red hydrogen peroxide/peroxidase assay kit (Invitrogen). Leaves (50mg) were homogenized in 0.5ml of 25mM phosphate buffer (pH 6.8). After centrifugation, 20 μl of the supernatant was incubated with 50 μl of reaction solution containing 0.2U ml^–1^ of horseradish peroxidase and 100 μM Amplex Red reagent (10-acetyl-3,7-dihydrophenoxazine) for 30min in the dark. Absorption was measured at 560nm. The H_2_O_2_ concentration was determined by comparison with standard solutions (0–10 μM) of H_2_O_2_.

### Determination of antioxidant enzymes and ASA, DHA, GSH, and GSSG

For the enzyme assays, 500mg of leaves was homogenized in 5ml of 25mM phosphate buffer (pH 7.8) containing 0.2mM EDTA, 2mM ascorbic acid, and 2% polyvinylpyrrolidone. The homogenate was centrifuged at 12 000*g* for 20min at 4 °C and the supernatant was immediately used for the determination of enzymatic activity. Superoxide dismutase (SOD), catalase (CAT), guaiacol peroxidase (GPX), and ascorbate peroxidase (APX) activities assayed as described previously (Xu *et al.* 2012). Ascorbic acid (ASA), dehydroascorbic acid (DHA), glutathione (GSH) and glutathione disulfide (GSSG) contents were extracted and determined as previously described (Xu *et al.*, 2012).

### Oxidative damage estimation

Trypan blue staining was performed to show cell death. Leaves submerged in trypan blue (1.25mg ml; Sigma) were heated in a boiling water bath for 2min. The leaves were then decolorized in boiling ethanol (95%) for 15min.

Leaf relative water content (RWC) was defined as: RWC=(*F*_w_ – *D*_w_)/(*T*_w_ – *D*_w_)×100%, where *F*_w_ represents fresh leaf weight, *T*_w_ represents turgid leaf weight, and *D*_w_ represents dry leaf weight.

For electrolyte leakage (EL) detection, the sample was boiled at 100 °C for 15min to achieve 100% EL after measuring electrical conductivity. The relative conductivity of plasma membranes was calculated based on the ratio of electrical conductivity before and after boiling.

Lipid peroxidation was estimated by measuring the malondialdehyde (MDA) level as described previously (Zhu *et al.*, 2014).

### Statistical analysis

Statistical analysis of the results from experiments with three or more mean values used a one-way analysis of variance as dictated by the number of main effects. The difference was considered to be statistically significant when *P*<0.05.

## Results

### Effects of BR levels on the alternative respiratory pathway

**Fig. 1.**
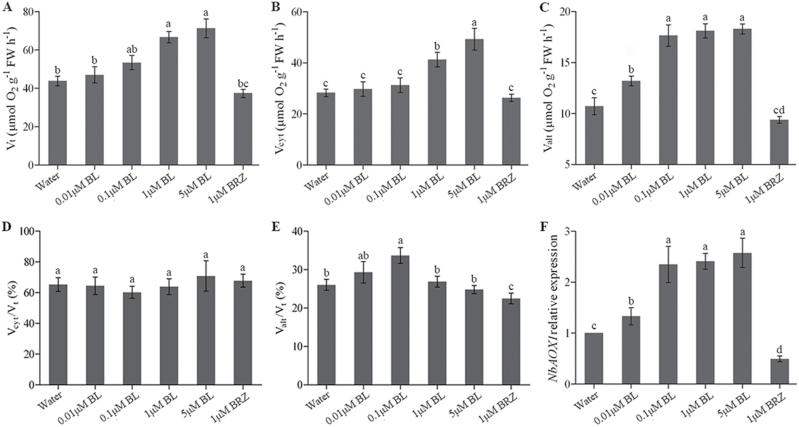
Effects of BR levels on the alternative respiratory pathway. *N. benthamiana* plants were sprayed with 0.01, 0.1, 1, or 5 μM BL solutions while the control plants were sprayed with distilled water or 1 μM BRZ. After pre-treatment for 24h, the sixth leaf of these plants was used for total respiration (*V*_t_) (A), cytochrome respiration (*V*_cyt_) (B), alternative respiration (*V*_alt_) (C), *V*_cyt_/*V*_t_ (D), *V*_alt_/*V*_t_ (E), and *NbAOX1* expression (F) measurement. Bars represent mean and standard deviation of values obtained from three biological repeats. Significant differences (*P*<0.05) are denoted by different lowercase letters. FW, fresh weight.

Total respiration rate (*V*_t_), the capacity of the alternative respiratory pathway (*V*_alt_) and cytochrome respiratory pathway (*V*_cyt_) were measured following treatment with water, different concentrations of BL, or BRZ for 24h. The results showed that there was a significant increase in *V*_t_ with increasing concentrations of BL treatment as compared with water pre-treated plants (Fig. 1A). Concomitantly, *V*_alt_ increased dramatically while *V*_cyt_ increased slightly in the presence of BL up to 0.1 μM, while a further increase in BL concentration from 0.1 to 5 μM had no significant increasing effects on *V*_alt_ (Fig. 1B, C). Consequently, the ratio of *V*_alt_/*V*_t_ increased and peaked at 0.1 μM BL (Fig. 1E), indicating an increased contribution of the alternative respiration pathway to total respiration. In contrast, the ratio of *V*_cyt_/*V*_t_ changed little in the presence of BL from 0.01 to 5 μM (Fig. 1D), suggesting small changes in the contribution of cytochrome respiration to total respiration under BL treatment. However, plants pre-treated with BRZ had a decrease in *V*_alt_ (Fig. 1C). We then analysed the transcript of *NbAOX1*, which has been shown be highly responsive to growth and stress conditions in *N. benthamiana* (Lee *et al.*, 2011). Similar to *V*_alt_, transcript levels of *NbAOX1* were upregulated in BL-treated plants but downregulated in BRZ-treated plants (Fig. 1F). Based on these results, 0.1 μM BL treatment was used in our subsequent experiments.

### Time course of BR-induced changes in alternative respiration and H_2_O_2_ production

**Fig. 2.**
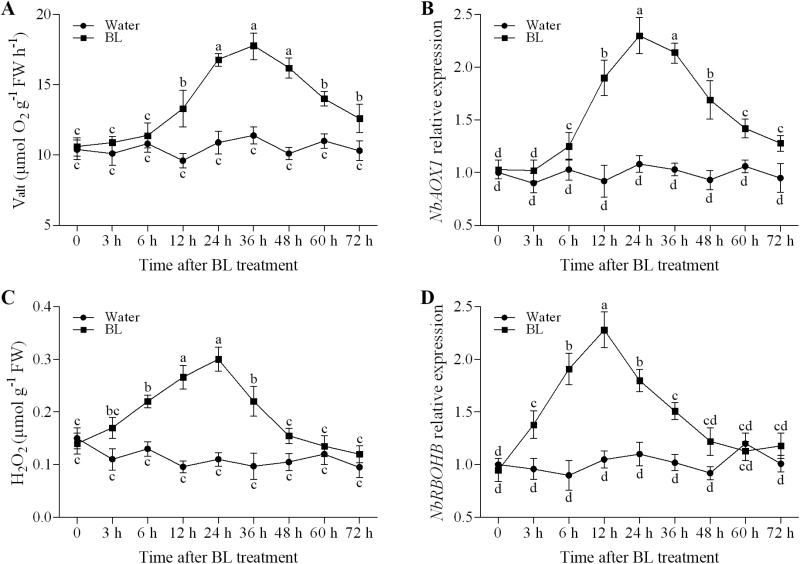
Time course of BR-induced changes in alternative respiration (*V*_alt_) (A), *NbAOX1* expression (B), H_2_O_2_ production (C), and *NbRBOHB* expression (D). *N. benthamiana* plants were sprayed with water or 0.1 μM BL, and the sixth leaf of each plant was harvested at the indicated time points for assays of alternative respiration, H_2_O_2_ content, and gene expression. Bars represent the mean and standard deviation of values obtained from three biological repeats. Significant differences (*P*<0.05) are denoted by different lowercase letters. FW, fresh weight.

In order to investigate the relationship between ROS and alternative pathway in BR signalling, we compared the time course of changes on *V*_alt_, H_2_O_2_ accumulation, and transcripts levels of *NbAOX1*, *NbRBOHA*, and *NbRBOHB* after BL treatment in *N. benthamiana*. As shown in Fig. 2, *V*_alt_ was not significantly altered during the first 6h but substantially increased at 12h and remained elevated up to 36h after BL treatment when compared with water-treated plants (Fig. 2A). Transcripts of *NbAOX1* increased at 6h, peaked at 24h, and then exhibited a gradual decline with extended time after BL treatment (Fig. 2B). In contrast, for H_2_O_2_ accumulation and transcripts of *NbRBOHB*, a significant increase occurred as early as 3h, and the maximum levels of H_2_O_2_ content and *NbRBOHB* expression were observed at 24 and 12h after treatment with BL, respectively (Fig. 2C, D). However, the expression of *NbRBOHA* was not significantly altered after BL treatment in comparison with *NbRBOHB* (Supplementary Fig. S1, available at *JXB* online). These results suggested that BRs induce ROS accumulation more rapidly than BRs induce the alternative pathway.

### ROS affect BR-induced alternative pathway capacity

**Fig. 3.**
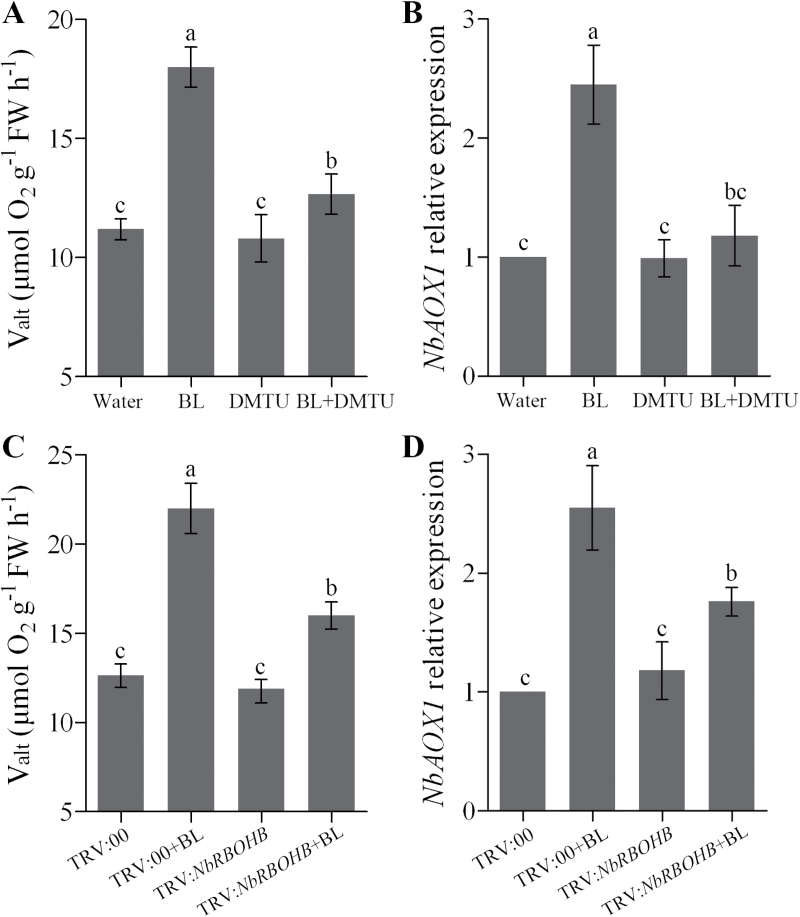
Involvement of H_2_O_2_ in the BR-induced alternative respiratory pathway. (A, B) Changes of alternative respiration (*V*_alt_) (A) and *NbAOX1* expression (B) in H_2_O_2_ scavenger DMTU pre-treated plants as influenced by 0.1 μM BL. *N. benthamiana* plants were treated with 5mM DMTU for 8h and then treated with 0.1 μM BL for another 24h. Single treatment of BL or DMTU was included as a control. (C, D) Changes in alternative respiration (C) and *NbAOX1* expression (D) in *NbRBOHB*-silenced plants as influenced by 0.1 μM BL. Bars represent mean and standard deviation of values obtained from three biological repeats. Significant differences (*P*<0.05) are denoted by different lowercase letters. FW, fresh weight.

To further investigate the effects of BR-induced H_2_O_2_ production on the BR-induced alternative respiratory pathway, *N. benthamiana* leaves were pre-treated with DMTU, an ROS scavenger, and then exposed to BL treatment. Pre-treatment with DMTU substantially reduced the BR-induced increases in *V*_alt_ and *NbAOX1* expression, whereas the single DMTU treatment had little effect on *V*_alt_ and *NbAOX1* expression when compared with water-treated plants (Fig. 3A, B). To further elucidate the role of NADPH oxidase (*RBOH*) genes in the regulation of the alternative pathway in BR signalling, we knocked down *NbRBOHA* and *NbRBOHB* using a TRV-based VIGS system in *N. benthamiana*. The downregulation of *NbRBOHA* and *NbRBOHB* in silenced plants was confirmed by real-time PCR after 12 d of infiltration (Supplementary Fig. S2, available at *JXB* online). Similar to DMTU treatment, BR-induced *V*_alt_ and *NbAOX1* expression were compromised in *NbRBOHB*-silenced plants in comparison with the control (TRV:00) (Fig. 3C, D) or *NbRBOHA*-silenced plants (Supplementary Fig. S3, available at *JXB* online). These results suggested that RBOHB-dependent H_2_O_2_ accumulation is required for the BR-induced alternative respiratory pathway.

### *Characterization of ROS-responsive motifs in the N. benthamiana* AOX1 *promoter*

**Fig. 4.**
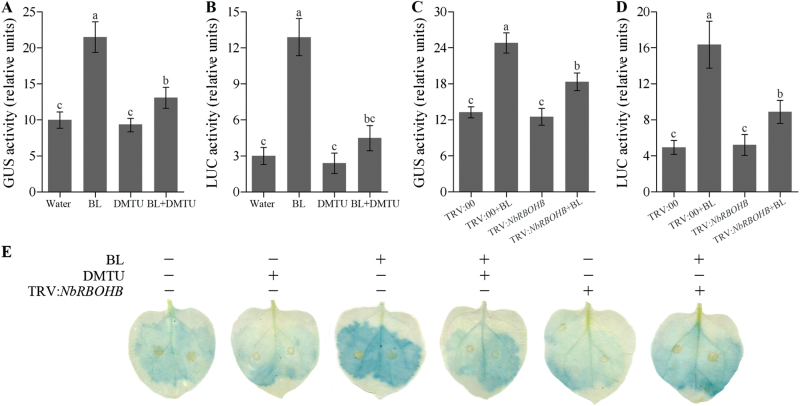
Effects of H_2_O_2_ on BR-induced *NbAOX1* promoter activity. (A, B) Relative GUS (A) or LUC (B) activity under the control of the *NbAOX1* promoter in 5mM DMTU pre-treatment plants as influenced by 0.1 μM BL. (C, D) Relative GUS (C) or LUC (D) activity under the control of the *NbAOX1* promoter in *NbRBOHB*-silenced plants as influenced by 0.1 μM BL. *N. benthamiana* plants were treated with 5mM DMTU for 8h and then treated with 0.1 μM BL for another 24h. Single treatment of BL or DMTU was included as a control. The CaMV 35S promoter was fused to GUS or LUC as a control for variation in transformation rate. Bars represent mean and standard deviation of values obtained from three biological repeats. Significant differences (*P*<0.05) are denoted by different lowercase letters. (E) GUS staining of *N. benthamiana* leaves transiently transformed with *NbAOX1* promoter fused to the GUS reporter gene as described in (A) and (C). Experiments were repeated three times with similar results. (This figure is available in colour at *JXB* online.)

As described above, ROS play an important role in BR-induced *NbAOX1* expression. In order to investigate the transcriptional regulatory linkage between *NbAOX1* gene and H_2_O_2_ accumulation induced by BRs, we analysed the *NbAOX1* promoter using the GUS and LUC reporter systems after transient transformation in *N. benthamiana* leaves. After BL treatment, GUS and LUC activities driven by the *NbAOX1* promoter were significantly upregulated when compared with water treatment. However, pre-treatment with DMTU substantially reduced the BR-induced *NbAOX1* promoter activity, whereas the pre-treatment had little effect in the absence of BL treatment (Fig. 4A, B). We also tested the transcriptional regulatory in *NbRBOHB*-silenced plants. Our results showed that BL treatment induced the *NbAOX1* promoter in TRV:00 plants obviously, whereas the treatment had little effect in TRV:*NbRBOHB* plants (Fig. 4C, D). GUS staining showed similar results (Fig. 4E).

**Fig. 5.**
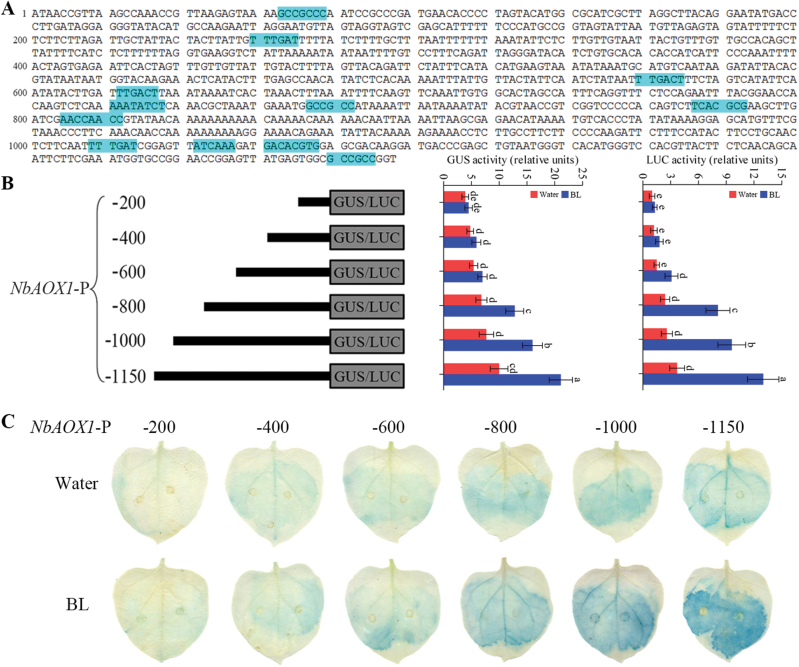
Characterization of ROS-responsive motifs in the *NbAOX1* promoter. (A) Sequence motifs common to the region 1.15kb upstream of *NbAOX1*. ROS-responsive motifs are indicated as light blue boxes. (B) A schematic representation of each construct is shown to the left along with the length of each upstream fragment in base pairs. The results from measurement of GUS or LUC activity for each construct are shown. The CaMV 35S promoter was fused to GUS or LUC as a control for variation in transformation rate. Bars represent mean and standard deviation of values obtained from three biological repeats. Significant differences (*P*<0.05) are denoted by different lowercase letters. (C) GUS staining of *N. benthamiana* leaves transiently transformed with each construct as described in (B), with or without 0.1 μM BL treatment for 24h. Experiments were repeated three times with similar results. (This figure is available in colour at *JXB* online.)

To further evaluate the role of ROS in BR-regulated *NbAOX1* transcription, ROS-response motifs in the *NbAOX1* promoter were searched with sequences indicated by Ho *et al.* (2008) and Petrov *et al.* (2012), and were found to be distributed primarily at the promoter downstream area (Fig. 5A). These ROS-response motifs were further confirmed by analysis of GUS and LUC activities driven by the *NbAOX1* promoter after transient transformation of *N. benthamiana* leaves. *NbAOX1* promoter activity sustained a gradual but mild increase with the increase in the promoter length before BL treatment. However, the more ROS-response motifs in the promoter fragments, the sharper increase in promoter activity derived by BL treatment (Fig. 5B). The result was also confirmed by GUS staining (Fig. 5C).

### BRs enhance AOX capacity in response to environmental stress

**Fig. 6.**
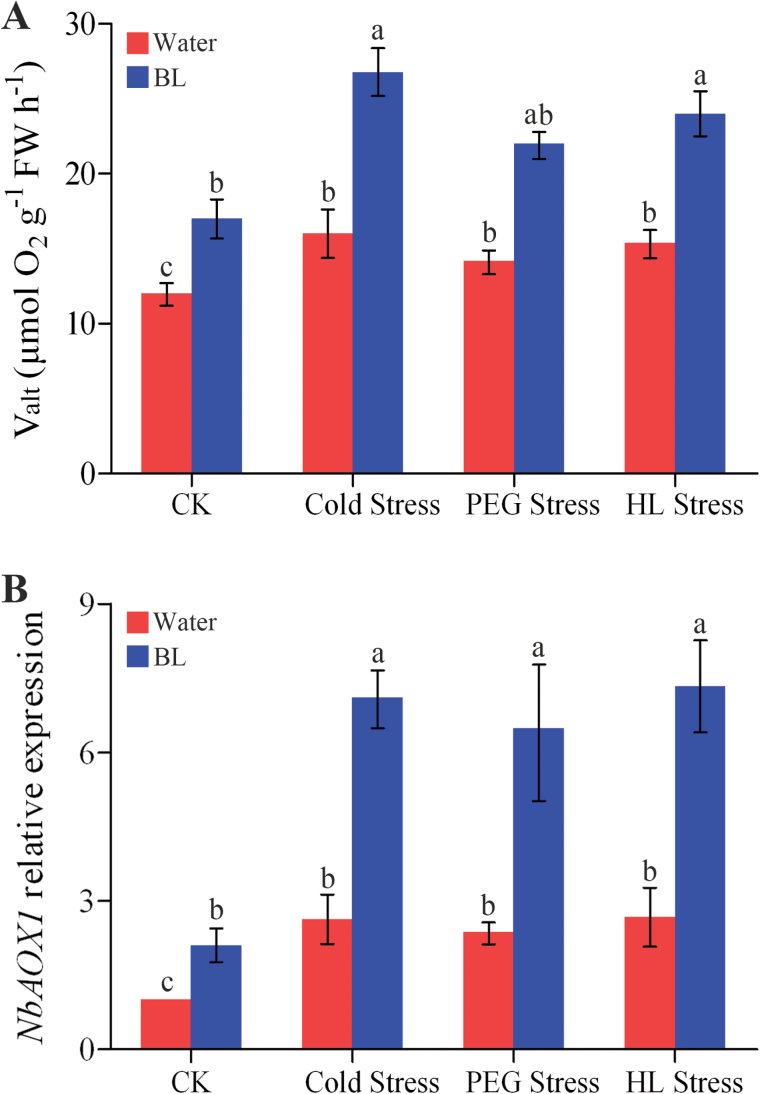
BRs induce AOX capacity in response to environmental stress. Alternative respiration (*V*_alt_) (A) and *NbAOX1* expression (B) in *N. benthamiana* plants after 12h of cold (4 °C), 16% PEG 6000, or HL (600 μmol m^–2^ s^–1^) stress. *N. benthamiana* plants were pre-treated with distilled water or 0.1 μM BL for 24h. Bars represent mean and standard deviation of values obtained from three biological repeats. Significant differences (*P*<0.05) are denoted by different lowercase letters. FW, fresh weight. (This figure is available in colour at *JXB* online.)

Abiotic stress and pathogen elicitors are known to stimulate the activity of the alternative pathway or at least increase *AOX* transcripts and/or protein levels (Van Aken *et al.*, 2009; Hanqing *et al.*, 2010). We also investigated whether BR treatment would further affect the alternative pathway capacity under cold, PEG, or HL stresses. The results showed that *V*_alt_ was higher in BL-treated plants than in control plants under all three types of stress for 12h (Fig. 6A). Consistent with the determined alternative respiration rate, the transcript level of *NbAOX1* was much higher in the BL-pre-treated plants than in the non-pre-treated control plants after 12h of stress (Fig. 6B). These data demonstrated that BRs enhance the alternative pathway in response to environmental stress.

### BR-induced AOX alleviates photosystem damage under stress conditions

**Fig. 7.**
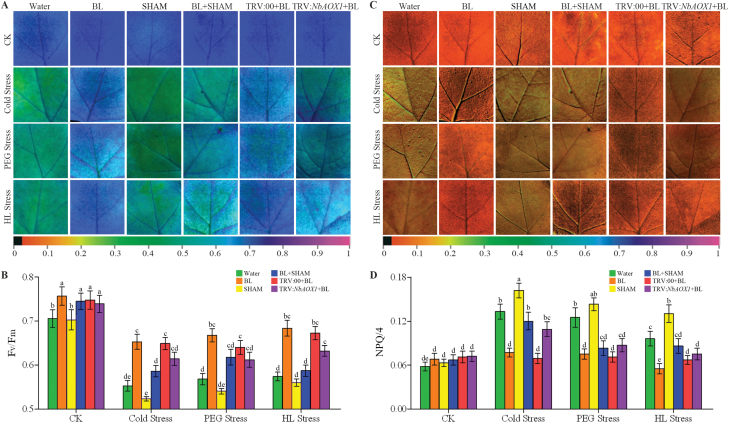
AOX alleviates photosystem damage in BR-induced stress tolerance. (A, C) Images of the maximum PSII quantum yield (*F*_v_ */F*_m_) (A) and NPQ/4 (C) in the eighth leaf of each *N. benthamiana* plant under cold (4 °C), 16% PEG 6000, or HL (600 μmol m^–2^ s^–1^) stress for 3 d. The alternative pathway was inhibited by 1mM SHAM pre-treatment or *NbAOX1* silencing in these BL-treated plants. (B, D) verage values of *F*_v_ */F*_m_ (B) and NPQ/4 (D) for the respective chlorophyll fluorescence images. Ten plants were used for each treatment and a picture of one representative leaf is shown. Bars represent mean and standard deviation of values obtained from six independent plants. Significant differences (*P*<0.05) are denoted by different lowercase letters. (This figure is available in colour at *JXB* online.)

To determine the role of the AOX pathway in BR-induced stress tolerance, we investigated the function of photosystem II (PSII) under stress conditions, as chlorophyll fluorescence quenching analysis has been proven to be a powerful and reliable method to assess changes in the function of PSII in the steady state of photosynthesis in response to different environmental stresses (Liu *et al.*, 2009). We first analysed the effects of SHAM, an AOX inhibitor, on BR-induced tolerance to cold, PEG, or HL challenge. As shown in Supplementary Fig. S4 (available at *JXB* online), SHAM (1mM) treatment inhibited 80% of alternative respiration when compared with the control. The results showed that in water-treated plants, the maximum photochemical efficiency of PSII in the dark-adapted state (*F*_v_ */F*_m_) was significantly lower than that in BL-treated plants under stress conditions (Fig. 7A, B). In contrast, NPQ was higher in water-treated plants but lower in BL-treated plants under the same stress conditions for 3 d (Fig. 7C, D). However, BR-induced tolerance to photo-oxidative stress, expressed as *F*_v_ */F*_m_ and NPQ, were largely inhibited if the plants were pre-treated with SHAM. To further determine the role of *NbAOX1* in BR-induced stress tolerance, we silenced *NbAOX1* using VIGS. The silencing effects on TRV:*NbAOX1* leaves were confirmed by comparing the expression levels with TRV:00 control leaves (Supplementary Fig. S2). Similar to SHAM pre-treatment, silencing of *NbAOX1* inhibited the alternative respiration pathway significantly (Supplementary Fig. S4). Importantly, BL treatment alleviated the significant decline of *F*_v_ */F*_m_ and increase of NPQ in TRV:00 plants under stress conditions, and these protective effects were again blocked in TRV:*NbAOX1* plants (Fig. 7). These results indicated that BR-induced AOX capacity plays a protective role in plant photosystems against environmental stress.

### BR-induced AOX alleviates oxidative damage and modulates ROS balance under stress conditions

**Fig. 8.**
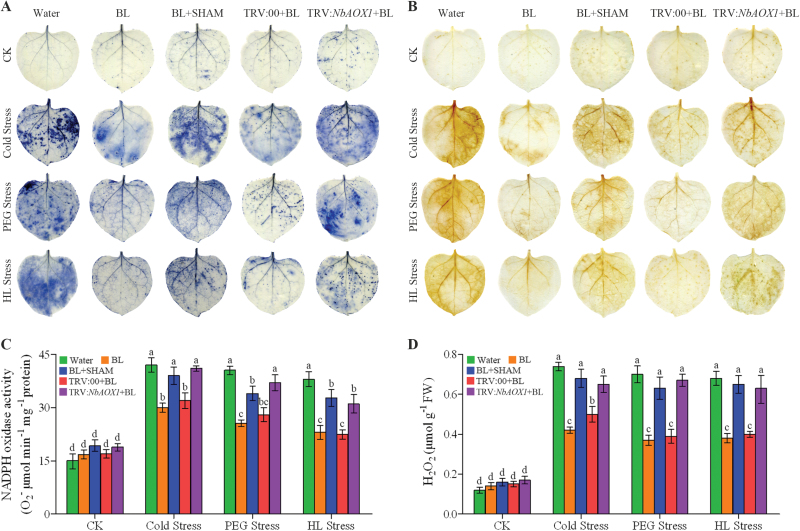
AOX modulates ROS balance in BR-induced stress tolerance. (A) Superoxide contents were detected by 0.5mg ml^–1^ of NBT staining. (B) Quantitative measurements of NADPH oxidase activity. (C) H_2_O_2_ levels were detected by 2mg ml^–1^ of DAB staining. (D) Quantitative measurements of H_2_O_2_ content. The alternative pathway was inhibited by 1mM SHAM pre-treatment or *NbAOX1* silencing in the BL-treated plants, and these plants were then challenged with cold (4 °C), 16% PEG 6000, or HL (600 μmol m^–2^ s^–1^) stress for 3 d. Experiments were repeated three times with similar results. Bars represent mean and standard deviation of values obtained from three biological repeats. Significant differences (*P*<0.05) are denoted by different lowercase letters. FW, fresh weight. (This figure is available in colour at *JXB* online.)

Plant responses to various types of stress are associated with the generation of ROS (Baxter *et al.*, 2013). We further detected the accumulation of superoxide and H_2_O_2_ using NBT and DAB staining procedures, respectively. Both procedures detected decreased staining in BL-treated leaves relative to that in water-treated leaves, although both increased after 3 d of stress. However, BR-decreased staining was largely inhibited in SHAM-pre-treated or TRV:*NbAOX1* leaves (Fig. 8A, B). We further analysed NADPH oxidase activity and H_2_O_2_ content. Similarly, in water-treated leaves, NADPH oxidase activity and H_2_O_2_ content were significantly higher than those in the BL-treated leaves under stress conditions, while these decreases were largely alleviated in SHAM-pre-treated or *NbAOX1*-silenced leaves (Fig. 8C, D).

**Fig. 9.**
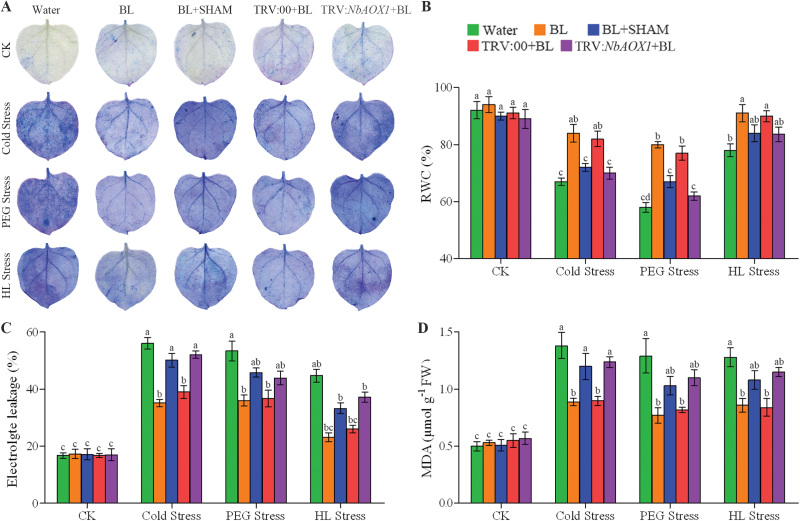
AOX alleviates oxidative damage in BR-induced stress tolerance. (A) Cell death was detected by staining with 1.25mg ml^–1^ of trypan blue. Experiments were repeated three times with similar results. (B) Quantitative measurements of RWC. (C) Quantitative measurements of EL. (D) Quantitative measurements of MDA content. The alternative pathway was inhibited by 1mM SHAM pre-treatment or *NbAOX1* silencing in the BL-treated plants, and these plants were then challenged with cold (4 °C), 16% PEG 6000, or HL (600 μmol m^–2^ s^–1^) stress for 3 d. Bars represent mean and standard deviation of values obtained from three biological repeats. Significant differences (*P*<0.05) are denoted by different lowercase letters. FW, fresh weight. (This figure is available in colour at *JXB* online.)

Other indices such as cell death, RWC, EL, and MDA content can also indicate the degree of damage in plants caused by environmental stress. Consistent with the ROS accumulation in Fig. 8, the BL-pre-treated *N. benthamiana* plants showed lower levels of cell death, EL, and MDA content compared with the control plants, although all of them increased under stress conditions (Fig. 9A–C). In addition, the RWC decreased significantly in the control plants after 3 d of stresses, but under the same stress conditions, BL-treated plants maintained higher RWC content than the control (Fig. 9D). However, these protective effects induced by BRs were again blocked in SHAM pre-treated or *NbAOX1* silenced plants (Fig. 9).

**Fig. 10.**
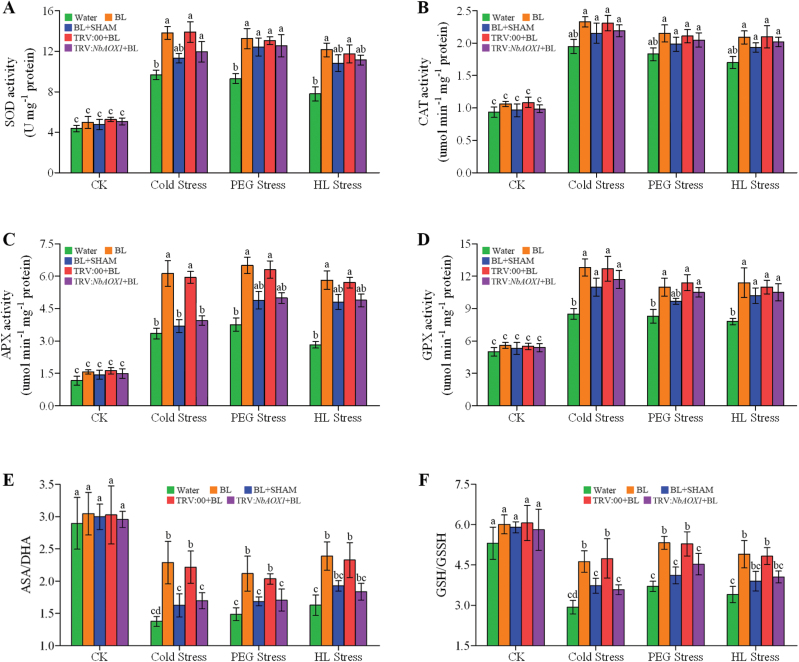
Changes in the activities of SOD (A), CAT (B), APX (C), GPX (D), ASA/DHA (E) and GSH/GSSG (F) in *N. benthamiana* plants under cold (4 °C), 16% PEG 6000, or HL (600 μmol m^–2^ s^–1^) stress for 3 d. The alternative pathway was inhibited by 1mM SHAM pre-treatment or *NbAOX1* silencing in these BL-treated plants. Bars represent mean and standard deviation of values obtained from three biological repeats. Significant differences (*P*<0.05) are denoted by different lowercase letters. (This figure is available in colour at *JXB* online.)

In general, the antioxidant defence machinery protects plants against oxidative stress damage. We next analysed changes in the activities of antioxidant enzymes (SOD, CAT, APX, and GPX) and ratios of GSH/GSSG and ASA/DHA after 3 d of stress. As shown in Fig. 10, the three types of stress conditions all resulted in significant increases in antioxidant enzyme activities and GSH/GSSG or ASA/DHA ratio, and these increases were significantly elevated with BL treatment. Interestingly, pre-treatment with SHAM or silencing of *NbAOX1* alleviated the BR-increased SOD, APX, and GPX activities, GSH/GSSG, and ASA/DHA ratios (Fig. 10A, C–F), suggesting more ROS accumulation and oxidative damage, while no significant difference was observed in CAT activities (Fig. 10B).

## Discussion

### BRs induce the alternative respiratory pathway in *N. benthamiana*

The role of plant mitochondria in stress resistance is of growing interest. Over the past decade, AOX has emerged as an important mitochondrial component in plant stress response. Most stressful conditions, including cold (Mizuno *et al.*, 2008), drought (Bartoli *et al.*, 2005), salt (Wang *et al.*, 2010) and aluminium (Panda *et al.*, 2008) stresses increase the *AOX* transcript abundance, the level of AOX protein, or the level of the alternative respiratory pathway. Many studies have revealed that AOX and the alternative respiratory pathway are also frequently induced during plant–pathogen interactions (Zhang *et al.*, 2012; Cvetkovska and Vanlerberghe, 2013). A number of plant hormones are known to be involved in AOX induction. It was reported that low concentrations of salicylic acid are effective at inducing *AOX* expression and alternative respiratory activity in tobacco (suspension cells and intact leaves) and soybean hypocotyls (Lennon *et al.*, 1997; Matos *et al.*, 2009). Jasmonic acid can strongly increase steady-state *AOX* transcription levels in sweet peppers and reduce the incidence of chilling injury (Fung *et al.*, 2004). Ethylene is closely associated with AOX induction under several stress conditions (Wang *et al.*, 2010; Xu *et al.*, 2012). In *Arabidopsis*, the *AtAOX1* promoter is upregulated directly by exogenous abscisic acid treatment, and the induction of *AOX1* expression is abolished in an *abi4* mutant (Giraud *et al.*, 2009). These reports suggest a connection between AOX and plant hormones. In the present study, we demonstrated that BRs could also induce the alternative respiratory pathway in *N. benthamiana*. Exogenously applied BL upregulated CN-resistant respiration and *NbAOX1* expression (Fig. 1). In addition, *NbAOX1* promoter activity was upregulated directly by exogenous BL treatment (Fig. 4). Our results imply a close correlation between BRs and the alternative respiratory pathway. To the best of our knowledge, this is the first report describing correlation of AOX induction with BRs in *N. benthamiana*.

### Involvement of ROS in BR-induced alternative pathway

ROS, especially H_2_O_2_ play an indispensable role in signal recognition and transduction in plant growth, development, and stress response. Recent studies show that BR-induced ROS accumulation enhances plant tolerance to abiotic stress (Xia *et al.*, 2009; Zhou *et al.*, 2014*a*). It is postulated that H_2_O_2_ may be a secondary messenger in the signal transduction pathway to induce alternative respiration (Neill *et al.*, 2002). In this study, we have provided evidence that BR-induced H_2_O_2_ production is necessary for the BR-induced alternative respiratory pathway. BR-induced ROS accumulation was more rapid than BR induction of the alternative pathway (Fig. 2). Our experimental results also showed that scavenging of H_2_O_2_ by pre-treatments with DMTU blocked BR-induced alternative respiration, *NbAOX1* expression, and *NbAOX1* promoter activity (Figs 3 and 4). Furthermore, we analysed ROS-responsive motifs in the *NbAOX1* promoter and performed deletion experiments, with the results indicating that ROS induced by BRs was required for *NbAOX1* transcriptional regulation (Fig. 5).

NADPH oxidase encoded by *RBOH* genes is a main source of BR-induced H_2_O_2_ accumulation (Zhang *et al.*, 2010; Zhou *et al.*, 2014*b*). In the present study, we observed that BRs increased transcript levels of *NbRBOHB* but not *NbRBOHA* significantly in *N. benthamiana* (Fig. 2D and Supplementary Fig. S1). We also found that BRs failed to increase alternative respiration, *NbAOX1* expression and *NbAOX1* promoter activity in *NbRBOHB*-silenced plants (Figs 3 and 4), suggesting *RBOHB*-dependent H_2_O_2_ production plays an important role in the BR-induced alternative respiratory pathway. In addition, AOX capability was not completely abolished by scavenging of H_2_O_2_ (Figs 3 and 4), suggesting that, besides the H_2_O_2_-dependent pathway, other unknown factors such as post-translational modification or the BR signalling pathway may also contribute to the BR-induced alternative pathway in *N. benthamiana*.

### AOX is involved in BR-induced stress tolerance

Currently, AOX is receiving considerable attention from plant scientists and is believed to play an important role in abiotic stress alleviation. Recent studies reveal that, besides their critical role in orchestrating growth and developmental processes, BRs are also implicated in plant responses to abiotic and biotic stresses (Nakashita *et al.*, 2003; Xia *et al.*, 2009, 2011). Similar to previous reports, our study showed that cold, PEG, and HL stresses induced alternative respiration and *NbAOX1* expression, and these increases were enhanced by BL treatment (Fig. 6). These results suggest that AOX is very likely to participate in BR-induced environmental stress tolerance.

ROS generation is a common cellular response during stressful conditions. On the one hand, the generation of ROS is necessary for signalling in individual stress responses. On the other hand, ROS overproduction causes oxidation damage to cellular components, which is a common destructive effect of abiotic stress. Time courses of the effects of BL on alternative respiration and H_2_O_2_ accumulation showed that BRs induced alternative respiration and ROS production mildly under normal growth conditions. Interestingly, when exposed to stress conditions, water-treated plants suffered a dramatic increase in H_2_O_2_ accumulation, forming a sharp contrast with BL-treated plants, which had a comparatively slight increase. However, BL-treated plants enjoyed a sharper increase in alternative respiration when compared with water-treated plants under stress conditions (Supplementary Fig. S5, available at *JXB* online). The enhancement of AOX by BL pre-treatment might play a key role in ROS avoidance under stress conditions. In general, AOX can function to prevent ROS formation from an over-reduced ubiquinone pool in plant mitochondria (Noctor *et al.*, 2007; Rasmusson *et al.*, 2009). It has been reported that AOX inhibition stimulates ROS production in plant mitochondria (Popov *et al.*, 1997; Møller, 2001), whereas overexpression of AOX results in lower ROS levels (Maxwell *et al.*, 1999). Moreover, some studies show that plants lacking AOX suffer greater damage under stress conditions (Van Aken *et al.*, 2009). Notably, our results showed that the inhibition of AOX activity by SHAM pre-treatment or *NbAOX1* silencing in BL-treated plants caused a sharp increase in ROS production under environmental stress (Fig. 8). Antioxidant enzymes can scavenge superfluous ROS caused by stress conditions and protect plants from oxidative damage. In the present study, we found that inhibition of AOX in BL-treated *N. benthamiana* plants had negative effect on the activities of SOD, CAT, APX, and GPX, and these plants showed lower ratios of ASA/DHA and GSH/GSSG under stress conditions (Fig. 10). It has been demonstrated that ASA and GSH play an important role in countering ROS production during biotic or abiotic stress (Xu *et al.*, 2012). Consequently, analysis of cell death, RWC, EL, and MDA contents showed that inhibition of the alternative pathway caused more damage in BL-treated plants (Fig. 9). These results demonstrate that BR-induced AOX capability can avoid superfluous ROS accumulation and protect plants from oxidative damage in response to environmental stress. It has been reported that SHAM is an inhibitor of peroxidases and xanthine oxidase (Able *et al.*, 2000). However, according to Bartoli *et al.* (2005), 1mM SHAM is sufficiently low to minimize possible side effects. Thus, the decreased protection caused by SHAM treatment is most likely a consequence of the inhibited alternative pathway.

The data presented here also suggest that the enhanced AOX activity by BRs contributes to protection of chloroplasts under stress conditions. In the present work, inhibition of AOX in plants with or without BR treatment resulted in lower levels of photochemical efficiency and dissipated more excitation energy by NPQ under stress conditions (Fig. 7), similar to a previous report (Giraud *et al.*, 2008). Previous findings reported that the mitochondrial AOX pathway may play an important role in the protection of plants against photo-inhibition by alleviating the inhibition of the repair of the photodamaged PSII through prevention of ROS formation (Zhang *et al.*, 2011), while inhibition of the AOX pathway leads to decreases in photosynthetic rate in plants (Yoshida *et al.*, 2006; Zhang *et al.*, 2010, 2014*b*). It is also believed that AOX is required for dissipation of excess photosynthetic reductant, particularly during stress conditions (Yoshida *et al.*, 2006). Thus, our results, together with previous studies, suggest that the enhancement of AOX by BRs might play an important role in balancing chloroplast-to-mitochondria electron transfer, and thus decrease ROS accumulation in *N. benthamiana*.

In conclusion, our experimental results indicate that RBOHB-dependent ROS production is required for BR-induced AOX capability. The enhanced AOX then contributes to ROS avoidance and the protection of photosystems under environmental stress. Our results clearly suggest that AOX is involved in BR-induced stress tolerance, and contribute to our understanding of the signalling pathway mediated by BRs in response to environmental stress.

## Supplementary data

Supplementary data are available at *JXB* online.

Supplementary Table S1. Primers used for construction of VIGS vectors and real-time PCR analysis

Supplementary Table S2. Primers used for NbAOX1 promoter analysis.

Supplementary Fig. S1. Time course of BR-induced changes in *NbRBOHA* expression.

Supplementary Fig. S2. Confirmation of the *NbAOX1*, *NbRBOHA* and *NbRBOHB* genes silencing in *N. benthamiana* plants.

Supplementary Fig. S3. Changes of alternative respiration (*V*_alt_) and *NbAOX1* expression in *NbRBOHA*-silenced plants as influenced by 0.1 μM BL.

Supplementary Fig. S4. Effects of SHAM pre-treatment or *NbAOX1* silencing on respiration rate in *N. benthamiana* plants.

Supplementary Fig. S5. Time course of alternative respiration (*V*_alt_) and H_2_O_2_ production under stress conditions.

Supplementary Data

## Abbreviations:

AOX: alternative oxidase
APX: ascorbate peroxidase
ASA: ascorbic acid
BL: brassinolide
BR: brassinosteroid
BRZ: brassinazole
CaMV: cauliflower mosaic virus
CAT: catalase
CN: cyanide
DAB: 3,3′-diaminobenzidine
DHA: dehydroascorbic acid
DMTU: dimethylthiourea
EL: electrolyte leakage
GPX: guaiacol peroxidase
GSH: glutathione
GSSG: glutathione disulfide
GUS: β-D-glucuronidase
HL: high light
LUC: luciferase
MDA: malondialdehyde
NBT: nitro blue tetrazolium
NPQ: non-photochemical quenching
PEG: polyethylene glycol
PSII: photosystem II
ROS: reactive oxygen species
RT-PCR: reverse transcription-PCR
RWC: relative water content
SHAM: salicylhydroxamic acid
SOD: superoxide dismutase
TRV: tobacco rattle virus
VIGS: virus-induced gene silencing.
