# Supplementary material for: The alternative respiratory pathway is involved in brassinosteroid-induced environmental stress tolerance in Nicotiana benthamiana
Source: J Exp Bot. 2015 Jul 14;66(20):6219–32. doi: 10.1093/jxb/erv328 (PMC4588879; doi:10.1093/jxb/erv328)
Supplement: Supplementary Data [file supp_66_20_6219__index.html]

The alternative respiratory pathway is involved in brassinosteroid-induced environmental stress tolerance in Nicotiana benthamiana — The alternative respiratory pathway is involved in brassinosteroid-induced environmental stress tolerance in Nicotiana benthamiana — Supplementary Data 

# The alternative respiratory pathway is involved in brassinosteroid-induced environmental stress tolerance in *Nicotiana benthamiana*

## Supplementary Data

Data files

- Supplementary Data - Supplementary Data
