# Supplementary material for: The alternative respiratory pathway is involved in brassinosteroid-induced environmental stress tolerance in Nicotiana benthamiana
Source: J Exp Bot. 2015 Jul 14;66(20):6219–32. doi: 10.1093/jxb/erv328 (PMC4588879; doi:10.1093/jxb/erv328)
Supplement: Supplementary Data [file supp_erv328_jexbot146985_file001.pdf]

Alternative respiratory pathway is involved in brassinosteroids induced environmental stress tolerance in Nicotiana benthamiana

Xing-Guang Deng, Tong Zhu, Da-Wei Zhang, and Hong-Hui Lin

Supplemental Files

Supplementary Table S1 Primers used for construction of VIGS vectors and real-time PCR analysis

Supplementary Table S2 Primers used for NbAOX1 promoter analysis

Supplementary Fig. S1. Time course of BRs induced changes in NbRBOHA expression. N. benthamiana plants were sprayed with water or 0.1 μM BL, the sixth leaf of each plant was harvested at the indicated time points for assays of gene expression. Bars represent mean and standard deviation of values obtained from three biological repeats. Significant differences ($P<0.05$) are denoted by different lowercase letters.

Supplementary Fig. S2. Confirmation of the NbAOX1, NbRBOHA and NbRBOHB genes silencing in N. benthamiana plants. Real-time PCR analysis was conducted using total RNA extracted from the eigth leaves of plants inoculated with Agrobacterium GV2260 carrying TRV-target genes and the corresponding non-silenced leaves of TRV:00 infected control plants. Bars represent mean and standard deviation of values obtained from three biological repeats.

Supplementary Fig. S3. Changes of alternative respiration (Valt) and NbAOX1 expression in NbRBOHA-silenced plants as influenced by 0.1 μM BL. Bars represent mean and standard deviation of values obtained from three biological repeats. Significant differences ($P<0.05$) are denoted by different lowercase letters. FW, fresh weight.

Supplementary Fig. S4. Effects of SHAM pretreatment or NbAOX1 silencing on respiration rate in N. benthamiana plants. Bars represent mean and standard deviation of values obtained from three biological repeats. FW, fresh weight.

Supplementary Fig. S5. Time course of alternative respiration (Valt) and H2O2 production under stress conditions. N. benthamiana plants were sprayed with water or 0.1 μM BL, 24 h later, the plants were exposed to cold (4 °C), polyethylene glycol (PEG) (16 % PEG 6000) or high light (HL) (600 μmol m-2 s-1) stress. The sixth leaf of each plant was harvested at the indicated time points for assays of Valt and H2O2 content. Bars represent mean and standard deviation of values obtained from three biological repeats. FW, fresh weight.

Supplementary Table S1 Primers used for construction of VIGS vectors and real-time PCR analysis

Gene	Accession number	F primer	R primer
NbAOX1-VIGS	KF367455	GGAGGATGGATCAAAGCAC	ATGGCAATAGCAGGAGCAG
NbRBOHA-VIGS	AB079498	GCCGGTGGACATCCGATACG	CGTCGGCGATCGAGATTCCG
NbRBOHB-VIGS	AB079499	AGAGCGTCGTACAGTGGTC	GTAGGAATTGGGCGTTTAT
NbAOX1	KF367455	TGAATGATAAGCAGCACGAT	TGACGGTCCAATAAGCAAA
NbRBOHA	AB079498	ACAAATGTCTAAGCCTCCC	GCTCCATAAGGTCCATCTAT
NbRBOHB	AB079499	AACAACCTCGGATACATTAT	TGTAAATAGACCAGCCATAA
NbACTIN	AY179605	ACTGATGAAGATACTCACA	CAGGATACGGGGAGCTAAT

Supplementary Table S2 Primers used for *NbAOX1* promoter analysis

Primers	Sequences (5'→3')	Modification
*NbAOX1-P*200 F	CG*GGATCC*ATAACCGTTAAGCCAAACCG	*BamHI*
*NbAOX1-P*200 R	AA*CTGCAG*AGAAAAATACTACTCTAACA	*PstI*
*NbAOX1-P*400 F	CG*GGATCC*ATAACCGTTAAGCCAAACCG	*BamHI*
*NbAOX1-P*400 R	AA*CTGCAG*AAAATTTGGGAATGATGGTG	*PstI*
*NbAOX1-P*600 F	CG*GGATCC*ATAACCGTTAAGCCAAACCG	*BamHI*
*NbAOX1-P*600 R	AA*CTGCAG*TGAATATGACTAGAAAGTCA	*PstI*
*NbAOX1-P*800 F	CG*GGATCC*ATAACCGTTAAGCCAAACCG	*BamHI*
*NbAOX1-P*800 R	AA*CTGCAG*CAAGCTTCGCGTGAAGACTG	*PstI*
*NbAOX1-P*1000 F	CG*GGATCC*ATAACCGTTAAGCCAAACCG	*BamHI*
*NbAOX1-P*1000 R	AA*CTGCAG*GTTGCAGGAAGTATGGAAAG	*PstI*
*NbAOX1-P*1150 F	CG*GGATCC*ATAACCGTTAAGCCAAACCG	*BamHI*
*NbAOX1-P*1150 R	AA*CTGCAG*ACCGGCGGCGCCACTCATAA	*PstI*

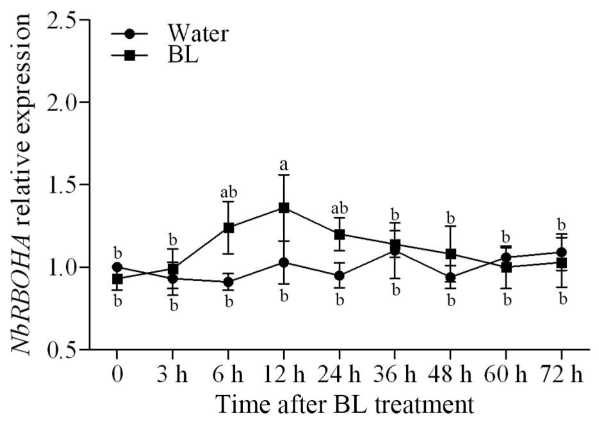
Water
BL
NbRBOHA relative expression
2.5
2.0
1.5
1.0
0.5
a
ab
ab
b
0
3 h
6 h
12 h
24 h
36 h
48 h
60 h
72 h
Time after BL treatment

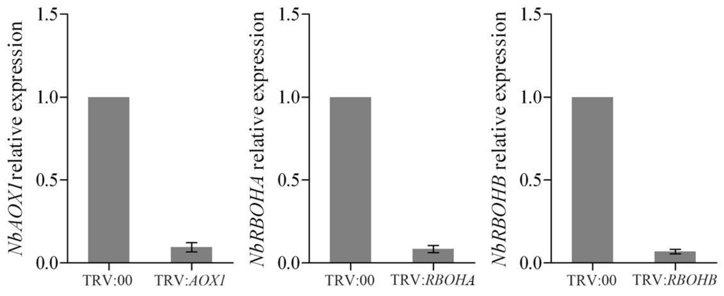

NbAOX1 relative expression
1.5
1.0
0.5
0.0
TRV:00
TRV:AOX1
NbRBOHA relative expression
1.5
1.0
0.5
0.0
TRV:00
TRV:RBOHA
NbRBOHB relative expression
1.5
1.0
0.5
0.0
TRV:00
TRV:RBOHB

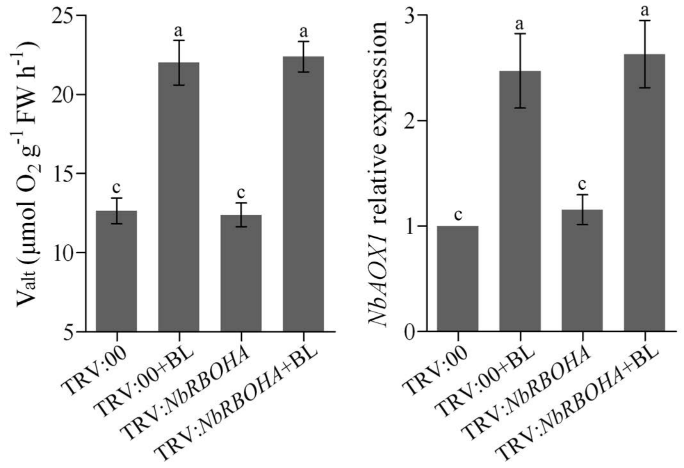

Valt (μmol O2 g-1 FW h-1)
25
20
15
10
5
a
a
c
c
TRV:00
TRV:00+BL
TRV:NbRBOHA
TRV:NbRBOHA+BL
NbAOX1 relative expression
3
2
1
0
a
a
c
c
TRV:00
TRV:00+BL
TRV:NbRBOHA
TRV:NbRBOHA+BL

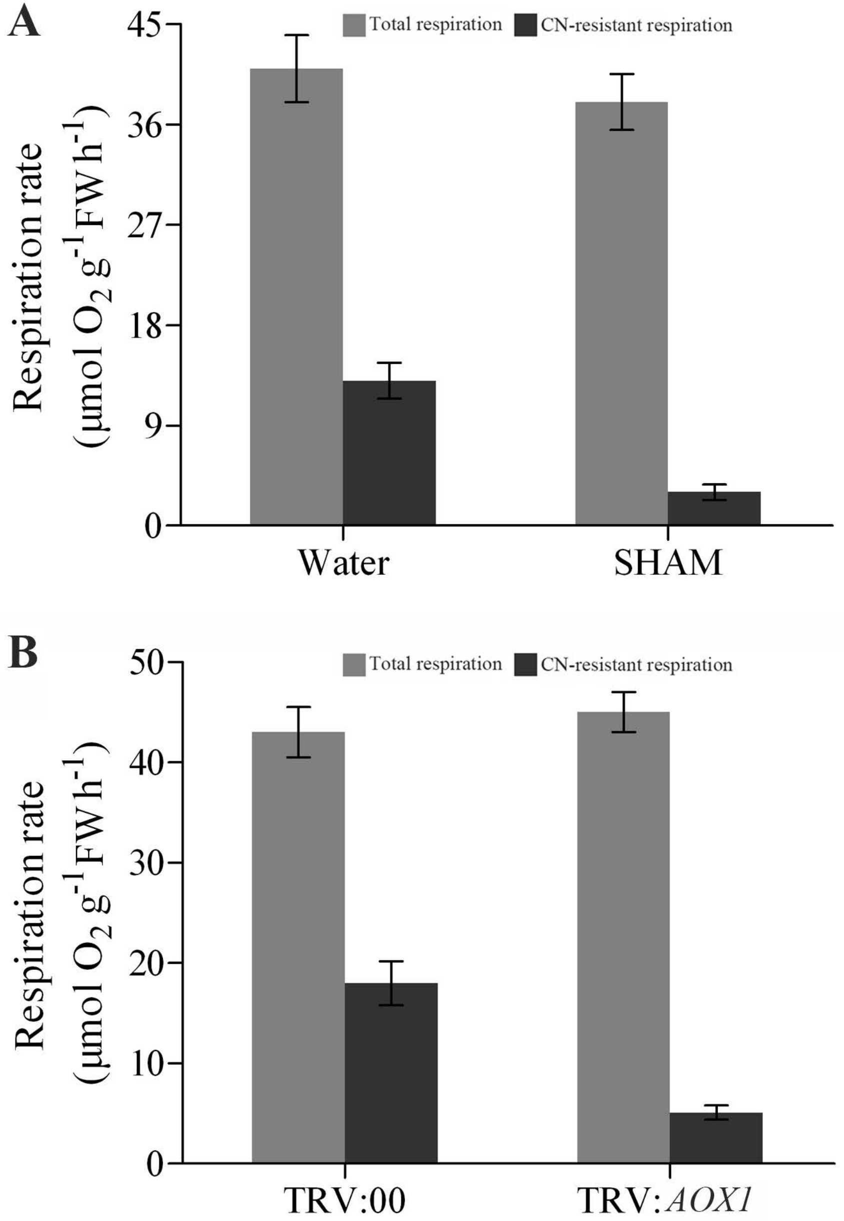
A
Total respiration
CN-resistant respiration
Respiration rate
(μmol O2 g-1 FW h-1)
45
36
27
18
9
0
Water
SHAM
B
Total respiration
CN-resistant respiration
Respiration rate
(μmol O2 g-1 FW h-1)
50
40
30
20
10
0
TRV:00
TRV:AOX1

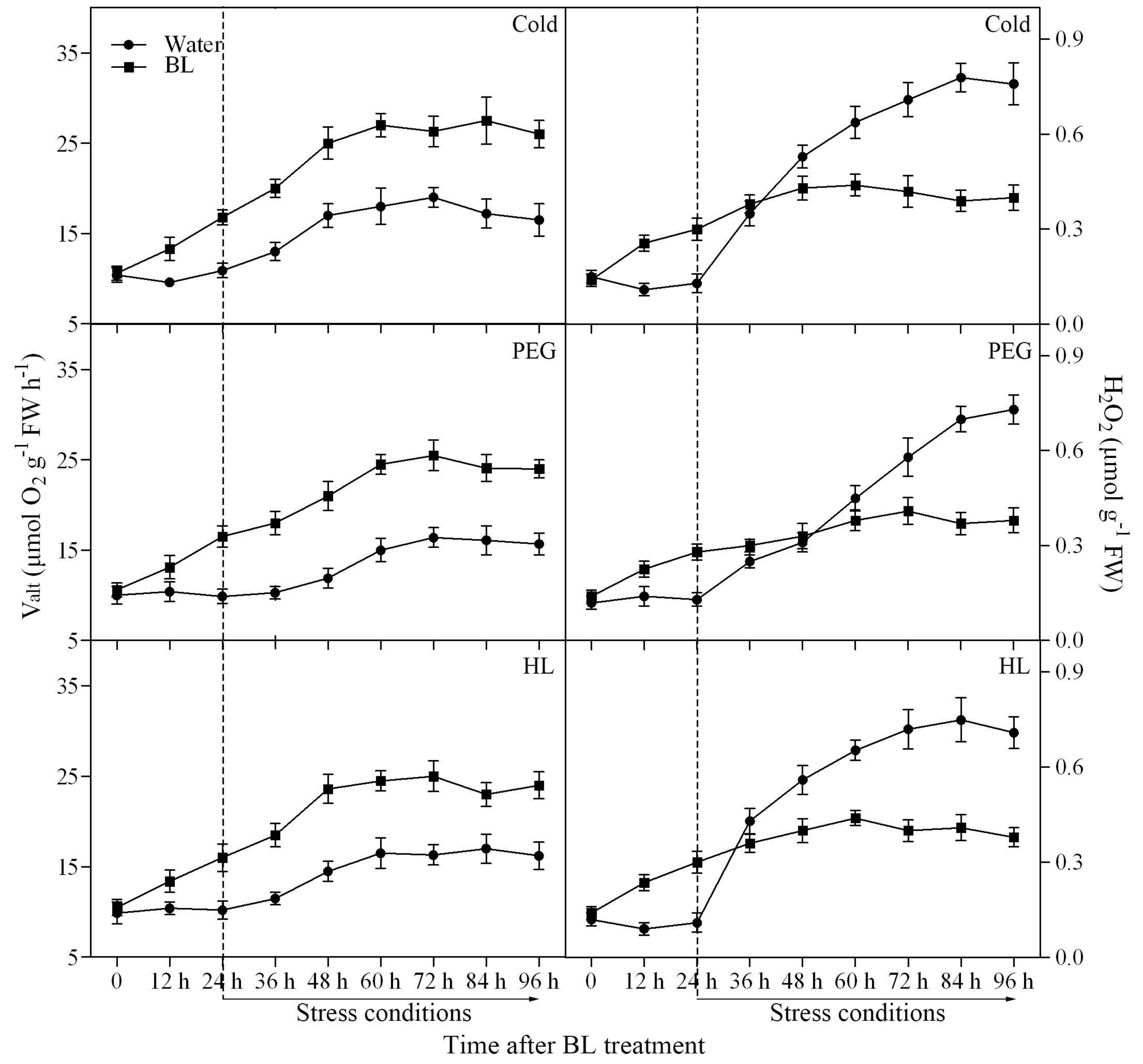

Cold
Cold
Water
BL
PEG
PEG
HL
HL
Valt (μmol O2 g-1 FW h-1)
H2O2 (μmol g-1 FW)
0
12 h
24 h
36 h
48 h
60 h
72 h
84 h
96 h
Stress conditions
Stress conditions
Time after BL treatment
